# Transcriptional response of honey bee (*Apis mellifera*) to differential nutritional status and *Nosema* infection

**DOI:** 10.1186/s12864-018-5007-0

**Published:** 2018-08-22

**Authors:** Farida Azzouz-Olden, Arthur Hunt, Gloria DeGrandi-Hoffman

**Affiliations:** 1 Kentucky State University, 400 East Main Street, Frankfort, KY 40601 USA; 2 Department of Plant and Soil Sciences, University of Kentucky, Lexington, KY 40546 USA; 3 USDA, ARS, Bee Research Center, 2000 East Allen Road, Tucson, AZ 85719 USA

**Keywords:** RNA-seq, Nutrition, *Nosema*, Honey bee, Immunity

## Abstract

**Background:**

Bees are confronting several environmental challenges, including the intermingled effects of malnutrition and disease. Intuitively, pollen is the healthiest nutritional choice, however, commercial substitutes, such as Bee-Pro and MegaBee, are widely used. Herein we examined how feeding natural and artificial diets shapes transcription in the abdomen of the honey bee, and how transcription shifts in combination with *Nosema* parasitism.

**Results:**

Gene ontology enrichment revealed that, compared with poor diet (carbohydrates [C]), bees fed pollen (P > C), Bee-Pro (B > C), and MegaBee (M > C) showed a broad upregulation of metabolic processes, especially lipids; however, pollen feeding promoted more functions, and superior proteolysis. The superiority of the pollen diet was also evident through the remarkable overexpression of vitellogenin in bees fed pollen instead of MegaBee or Bee-Pro. Upregulation of bioprocesses under carbohydrates feeding compared to pollen (C > P) provided a clear poor nutritional status, uncovering stark expression changes that were slight or absent relatively to Bee-Pro (C > B) or MegaBee (C > M). Poor diet feeding (C > P) induced starvation response genes and hippo signaling pathway, while it repressed growth through different mechanisms. Carbohydrate feeding (C > P) also elicited ‘adult behavior’, and developmental processes suggesting transition to foraging. Finally, it altered the ‘circadian rhythm’, reflecting the role of this mechanism in the adaptation to nutritional stress in mammals.

*Nosema*-infected bees fed pollen compared to carbohydrates (PN > CN) upheld certain bioprocesses of uninfected bees (P > C). Poor nutritional status was more apparent against pollen (CN > PN) than Bee-Pro (CN > BN) or MegaBee (CN > MN). *Nosema* accentuated the effects of malnutrition since more starvation-response genes and stress response mechanisms were upregulated in CN > PN compared to C > P. The bioprocess ‘Macromolecular complex assembly’ was also enriched in CN > PN, and involved genes associated with human HIV and/or influenza, thus providing potential candidates for bee-*Nosema* interactions. Finally, the enzyme Duox emerged as essential for guts defense in bees, similarly to *Drosophila*.

**Conclusions:**

These results provide evidence of the superior nutritional status of bees fed pollen instead of artificial substitutes in terms of overall health, even in the presence of a pathogen.

**Electronic supplementary material:**

The online version of this article (10.1186/s12864-018-5007-0) contains supplementary material, which is available to authorized users.

## Background

The European honey bee (*Apis mellifera*) is primarily reared for honey production, but its benefits are substantially higher considering it is the most important pollinator of crops [1]. Since 2006, US beekeepers have experienced yearly colony losses of up to 45% [2–4]. These losses, which are attributed to different stressors or stressor combinations [5, 6], extend to native bee species [7, 8]. Some stressors garnering special interest are pathogens [9, 10], climate change [11], limited dietary diversity [11], habitat loss [12, 13], pesticides [14–17], pathogen-pesticides synergy [18, 19] and disease-malnutrition synergy [20–22].

Colony survival depends on the availability of pollen (proteins, lipids, and micronutrients) [23] and nectar (carbohydrates). Not all pollen species have adequate nutritional composition, however [24]; hence monocultures may impose strong dietary constraints that can have harmful effects on bee health [25]. In fact, pollen intake influences lifespan [26] and several health indicators including physiological metabolism [27], immunocompetence [28], disease tolerance [22, 29] and pesticides resistance [30]. Monoculture hazards also encompass possible natural toxins in nectar or pollen that might be consumed in harmful concentrations [11].

To offset pollen shortages, beekeepers feed bees readily available and affordable plant-based protein substitutes [31]. However, the nutritional value of these commercial diets is unclear, as they performed similarly to pollen in one study [32], but were nutritionally poor or unpalatable in other studies [33–38]. Also, the long-term effectiveness of these diets on the colony health is unknown [11], prompting predictive efforts through mathematical modeling [39].

Throughout evolution, animals, facing starvation, have developed fitness traits to conserve energy and preserve organismal homeostasis [40]. *Drosophila melanogaster* enters diapause, a state of reproductive quiescence, arrested development, and extended lifespan. These reversible changes reveal immense phenotypic plasticity, which physiologically may reflect a tradeoff between programs geared toward growth and reproduction versus extended survival [41]. The pathway insulin/Igf-like signaling (IIS) is a key regulator of such processes. The IIS system, which inactivates a gene from the FOXO family that regulates metabolism and stress responses [42], is interrelated with the Target Of Rapamamicin (TOR) pathway [43]. The TOR cascade is mainly stimulated by amino acid abundance [44], and responds by upregulating translation to promote growth.

Like malnutrition, to offset metabolic cost, the immune response necessitates energy conservation by tradeoffs with reproduction and development [45]. The immune cost also lowers tolerance to additional stressors such as starvation [46] and, similarly, poor nutrition negatively impacts disease resistance [47, 48]. This nutrition-immunity interdependence led to the emergence of the study of “nutritional immunology”, which investigates dietary compositions for an optimized defense response [49]. In addition to specific nutrients molding the immune response [50, 51], another layer of complexity was recently added to this relationship when genetics was shown to play a role [52].

Because bees are facing increasing malnutrition and disease threats, it has become imperative to elucidate which of their organismal mechanisms are influenced by these stressors. To our knowledge, there are few studies of how pollen feeding influences the transcriptome of the honey bee, and no respective studies of artificial substitutes. Filling this knowledge gap can provide clues on the nutritional values of these diets and, possibly, on their long-term effectiveness on colony health. In addition, one of the most threatening pathogens to bee health is the midgut parasite *Nosema ssp.* [53, 54] from the group of microsporidia. Because these parasites have limited capacity for manufacturing ATP, and lack most primary metabolite genes [55], they impose a high metabolic cost on the host by appropriating these substrates from the host cell [56, 57]. However, although *Nosema* effects are expected to be exacerbated in the metabolically stressed malnourished bees, research results have lacked consistency in this regard [29, 58–61]. Thus, in this work we investigated the effects of pollen and pollen substitutes on the transcriptional response in healthy bees, but also in *Nosema-*infected bees.

## Methods

### Diets and *Nosema* trials

European honey bee colonies from the apiary of Carl Hayden Bee Research Center (USDA-ARS Tucson, AZ) were randomly sampled for frames of sealed brood which were placed in an environmental room kept at 34 °C, 30–40% humidity atmosphere to produce newly emerged bees. Within 10 h after emergence, the bees were collected and distributed into 24 cages with 100 bees per cage. All cages were provided ad libitum with carbohydrates (30% sucrose solution) and water. For diet treatments, the cages were organized in 4 groups of 6 cages. Three groups were fed ad libitum with proteinaceous diets (rich diets), which are respectively, pollen collected in the Sonoran Desert by colonies in the spring (P), Bee-Pro (B) and MegaBee (M). The fourth group received exclusively carbohydrates to constitute the control (C) also referred to as poor diet, hereafter. To create the diet/*Nosema* treatments PN, BN, MN, and CN, at day-7, for each diet, 3 cages were randomly selected and provided with a 60% sucrose feeding solution containing 10^5^ spores/bee of *Nosema apis* inoculum and were continued on the same diet as prior to infection for the remaining duration of the trial. The uninfected cages were maintained *Nosema*-free and were used to create the groups, hereafter, referred to as healthy or uninfected diet treatments P, B, M and C. Such design, thus, yielded 4 diet/no-*Nosema* and 4 diets/*Nosema* treatments, each containing 3 biological replicates (3 cages). Experimentation was terminated on day-14, on which bees were flash frozen and stored at − 80 °C for subsequent mRNA and hemolymph extractions.

### Protein analysis and RNA-seq libraries preparation

To determine the protein concentrations of the hindgut and hemolymph, 3 bees were randomly selected from each treatment, and their abdomens were collected for processing according to protocols published elsewhere [62, 63]. To perform the transcriptome profiling, for each treatment, 12 bees per biological replicate (cage) were randomly chosen, and their heads discarded. The abdomens were divided into 4 groups of 4 abdomens that were collectively subjected to homogenization in Trizol, followed by RNA extraction using RNeasy kit (Qiagen). Subsequently, all three RNA pools were equally combined into a larger single RNA bioreplicate. The three biological RNA samples, thus obtained per treatment, were used to prepare RNA-Seq libraries as described elsewhere [64], which were sequenced on the Illumina platform.

### Analysis of sequenced data

Sequencing data were analyzed using CLC Genomics Workbench 7.5.1 (Qiagen). First, sequences were preprocessed for duplicate removal and demultiplexed into separate libraries representing the various replicates. The bee genome Amel 4.5_scaffolds was used as a reference for mapping the reads. Mapping options were set at mismatch cost 2, insertion cost 3, depletion cost 3, length fraction 0.5, similarity fraction 0.8, and gene expression value set to RPKM [65]. Differential expression analysis was performed with the ‘Transcriptomics Analysis’ toolbox, and comprised ‘experiment set-up’, where treatments pairs were analyzed with the option ‘All group pairs’. This setting uses the Wald test, and reports the expression mean of each gene with fold change between the treatment pair. Expression values were normalized using the options ‘by totals’ and ‘state numbers in read 1,000,000’. The normalized values were transformed using “Add a Constant” set at the value ‘1’. In order to identify the differentially expressed genes (DEGs) between a pair of treatments, a t-test was performed on the transformed values for each mapped gene, and DEGs were filtered based on *p*-value cutoff *p* < 0.05 and fold change cutoff FC ≥ |1.5|. *Drosophila* homologs were identified using BioMart (Ensembl) and the Hymenoptera genome database [66], and used for gene ontology analysis (GO) to uncover significantly enriched bioprocesses [67] and pathways (KEGG). REVIGO [68] and GO browser (GO tree) were used to remove redundant GO-terms resulting from the functional analysis. Significance of the number of genes overlapping between DEG lists was determined by calculating a ‘representation factor’ [69]. The overlap was further examined for concordance in the direction of regulation utilizing contingency tables on which a chi-square test followed by Yates correction were performed using R environment.

### RT-qPCR of selected genes

Gene selection for qPCR testing was primarily based on the RNA-seq results, but also on their role in the nutritional or immune responses. We examined vitellogenin (Vg) expression because of its importance as a storage protein accumulated under rich nutritional status. In fact, Vg is a proven marker gene that is responsive to rich diet and is overexpressed under rich nutritional status. Therefore Vg is especially relevant to test whether natural and commercial diets differ in their nutritional value. We also selected NADPH dual oxidase (Duox) because, to our knowledge, it was never linked to honey bee gut defense, while it was recently shown as central in *Drosophila* gut immunity (see section: effects of nutrition on immunity).

Extracted RNA was first treated with DNase 1 to eliminate contaminating genomic DNA using the GenElute binding columns (Sigma). Following reverse transcription using the cDNA Synthesis Kit (Sigma), qPCR was performed in a reaction of 10 μl total volume containing 2× Brilliant II SYBR Green ReadyMix (5 μl), 0.4 μM of each primer, and cDNA sample (2 μl of 1/10 dilution). The genes e1f11 and Rp49 were used for efficiency correction, and primer sequences for each gene were as previously published (Vg: [70], Duox: [71], e1f11: [27], Rp49: [72]). The thermal reactions consisted of 40 cycles with the annealing step set at 50 °C for Duox, 51 °C for Rp49, 54 °C for E1f111, and 55 °C for Vg. All healthy treatments and all infected treatments were, respectively, evaluated for Vg and Duox expression. Using R environment, results were analyzed, first by testing for normality (Shapiro test), then assessing equality of variance (variance test). Subsequently, relative expression levels were tested for differences in significance with t-test and Wilcoxon test, respectively, when data were normally and non-normally distributed.

## Results

### Protein content

The soluble protein concentration of pollen was 3 and 5 times higher than Bee-Pro, and MegaBee, respectively (Table 1). Regarding protein digestibility, a significant diet effect was observed in healthy bees and in *Nosema*-infected bees, with significantly higher concentration of undigested proteins in the hindgut when bees were fed MegaBee or Bee-Pro, compared with pollen (Table 2). *Nosema* infection did not significantly alter hindgut protein content for any of the three diets (Table 2). Similarly to the hindgut, protein titer of the hemolymph showed significant between-diets differences in uninfected and *Nosema*-infected bees. Pollen and Bee-Pro feeding induced higher protein titers, contrary to the lower level (indistinguishable) under MegaBee and carbohydrates feeding (Table 2). As with the digestibility assay, within-diets, *Nosema* had no effect on the protein levels in the hemolymph.

**Table 1:** Means (μg/ml) of soluble proteins of Sonoran Desert pollen and pollen substitutes

Diet	ANOVA			Tukey test			
	Mean	F	p-Val	Comparison	Q statistics	p-Val	Tukey rank
Pollen (P)	758.7	139.59	0.000	P vs. B	18.6997	0.0010053	1
Bee-Pro (B)	280.3			P vs. M	21.8737	0.0010053	2
MegaBee (M)	199.1			M vs. B	3.1740	0.1410405	2

**Table 2:** Proteins content (μg/ml) of the hindgut and hemolymph in bees with and without *Nosem*a fed different diets

Hindgut								Hemolymph					
Diet	t-test within-diet			ANOVA between-diet (N+)		ANOVA between-diet (N-)		t-test within-diet			ANOVA between-diets		
	N-	N+	p-val	Statistics	Rank	Statistics	Rank	N-	N+	p-val	Mean	Statistics	Rank
											(within diet)		
P	0.258	0.216	0.149	F = 341 *p* = 0.000	3	F = 25.32 *p* = 0.001	3	519.2	569.3	0.595	544.2	F = 8.69 p = 0.001	1
B	0.637	0.527	0.258		2		2	463.9	529.3	0.593	496.6		1
M	0.869	0.913	0.702		1		1	333.6	311.5	0.703	322.6		2
C	–	–	–		–		–	351.9	248	0.341	299.9		2

Diet rank was determined using Tukey test, N- and N+, respectively, describe treatments without and with *Nosema*

### RNA-seq analysis

#### Sequencing statistics

The sequencing generated 2 datasets, Diets_no_*Nosema* and Diets_*Nosema*, each containing 3 libraries per diet. The two datasets consisted of 34,183,883,593 sequenced nucleotides from 338,454,293 reads that passed the initial quality control. When mapped to the honey bee genome, Diets_no_*Nosema* generated 45,720,190 uniquely mapped sequences while Diets_*Nosema* generated 35,707,804 uniquely mapped sequences. In terms of genes queried by these reads, the Diets_no-*Nosema* and Diets_*Nosema* samples included 32,721,777 and 26,453,873 uniquely-mapped reads that mapped to 15,314 honey bee genes (Table 3).

**Table 3:** Statistics of RNA-seq mapped sequences by type

	Uniquely mapped	Fraction	Non-specifically mapped	Fraction	Mapped	% of total mapped
a) Twelve libraries of diet/healthy-bees						
Total gene	32,721,777	0.99	297,597	0.01	33,019,374	71.28
Intergenic	12,998,413	0.98	302,819	0.02	13,301,232	28.71
Total	45,720,190	0.99	600,416	0.01	46,320,606	100
b) Twelve libraries of diet/infected-bees						
Total gene	26,453,873	0.99	282,617	0.01	26,736,490	73.87
Intergenic	9,253,931	0.98	205,394	0.02	9,459,325	26.13
Total	35,707,804	0.99	488,011	0.01	36,195,815	100

#### Genome-wide regulation

The results of the gene expression analysis are summarized in Table 4. In healthy bees, pollen feeding compared to carbohydrates (P vs. C) instigated more differential transcription than Bee-Pro (B vs. C) and MegaBee (M vs. C), while the latter diets were similar in that regard. *Nosema* stress sharply accentuated global differential regulation under pollen (PN vs. CN), but affected slightly bees fed Bee-Pro (BN vs. CN) and MegaBee (BN vs. CN). A tendency to global upregulation was evident in conditions of malnutrition, and heightened when combined with *Nosema*. The upregulation under malnutrition was wider in the comparisons to pollen treatments (C > P and CN > PN), uncovering larger numbers of DEGs than the comparisons to the substitutes. This trend holds true for a select subset of known genes recorded in at least 2 treatments (Additional file 1, Fig. 1). In healthy bees, overexpression under rich nutrition, especially pollen (P > C), was less marked than overexpression under carbohydrates only diet (C > P). *Nosema* infection did not affect the number of upregulated genes when bees were fed pollen (PN > CN), however it exerted a severe inhibitory effect in bees fed Bee-Pro (BN > CN) or MegaBee (MN > CN).

**Table 4:** Genes differentially transcribed in bees with or without *Nosema* fed rich diets versus carbohydrates

Treatment	Comparison	↑ Genes	↑*Drosophila* orthologs	↓ Genes	↓ *Drosophila* orthologues	Total *A. mellifera* genes
Pollen/*Nosema* Pollen/no-*Nosema*	PN vs. CN	52	40	1489	906	1541
	P vs. C	50	42	577	306	627
MegaBee/*Nosema* MegaBee/no-*Nosema*	MN vs. CN	91	49	231	134	322
	M vs. C	208	113	89	48	297
Bee-Pro/*Nosema* Bee-Pro/no-*Nosema*	BN vs. CN	18	13	219	92	237
	B vs. C	148	90	135	80	283

Numbers of genes regulated by rich diet feeding. Differential expression in healthy bees fed pollen (P), Bee-Pro (B) or MegaBee (M) is assessed against carbohydrates only diet (C). Respectively, the same diet treatments in *Nosema*-infected bees are referred to as PN, BN and MN, which differential expression is considered against carbohydrates/*Nosema* (CN). The up and down arrows denote genes that are up- or downregulated through RNA-seq analysis; the numbers of known *Drosophila* orthologs are also described

**Fig. 1:**
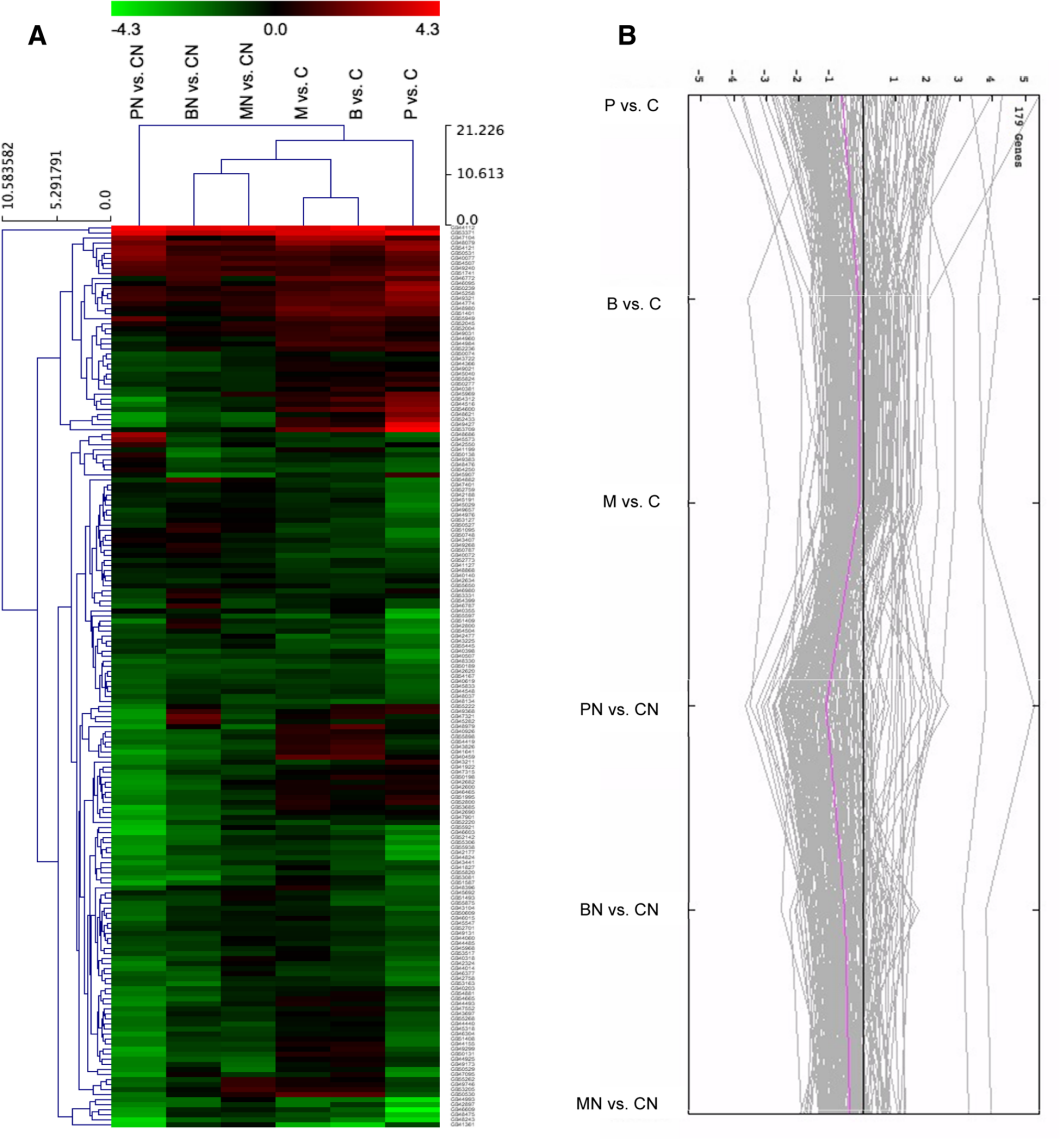
Hierarchical cluster analysis of functionally known DEGs. **a** Select number of genes figuring at list in 2 treatments. PN vs. CN, BN vs. CN, and MN vs. CN are, respectively, pollen, BeePro and MegaBee diet effects compared to carbohydrates in presence of Nosema. P vs. C, B vs. C and M vs. C are the same effects, respectively, compared to carbohydrates in absence of Nosema. **b** Expression graph corresponding to the select DEGs represented in the hierarchical cluster analysis

### Gene ontology analysis

#### Effects of nutrition in healthy bees

**Fig. 2:**
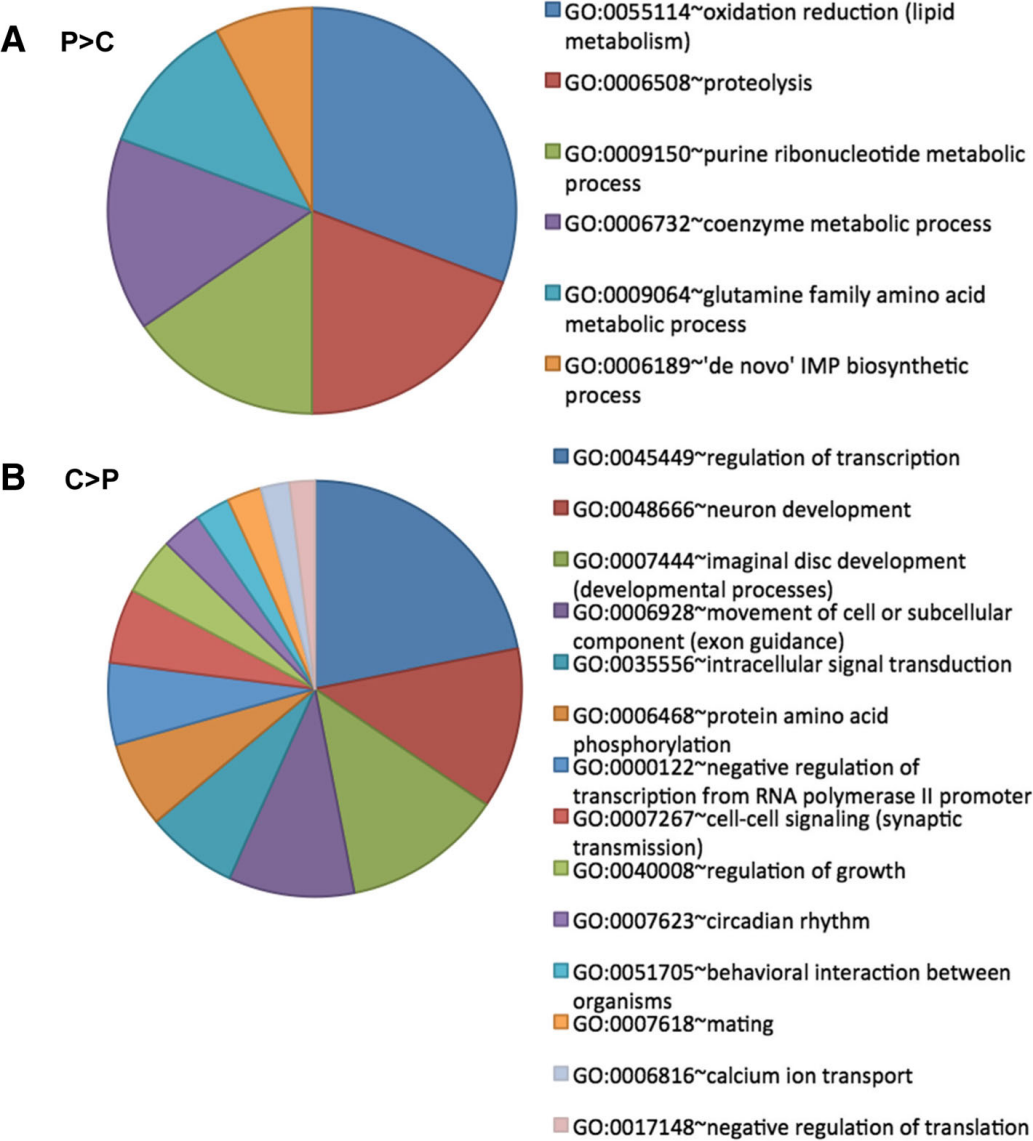
Upregulated bioprocesses under differential nutritional status. **a** GO-terms upregulated under pollen feeding compared to carbohydrates in healthy bees (P > C). **b** GOterms upregulated under poor diet feeding, carbohydrates, compared to pollen in healthy bees (C > P). Pie chart shows the percentage of genes involved in the GO terms

The GO analysis of expression upregulation revealed that healthy bees fed a rich diet (P > C, B > C and M > C) exhibited a stimulated metabolism, albeit Bee-Pro effect was slightly above significance cutoff (‘metabolic pathways’, *p* = 0.065). Although, pollen feeding (P > C) upregulated fewer genes, it affected more bioprocesses (Fig. 2a) than MegaBee (M > C) and Bee-Pro (B > C) (Additional file 2).

Comparison of carbohydrates feeding to pollen (C > P) showed more altered bioprocesses (Fig. 2b) than comparison to Bee-Pro (C > B) or MegaBee (C > M) (Additional file 3). Five genes associated with response to starvation (CG7728, CG9107, Atg6, CG2972 and Ak6) were upregulated in C > P, while only one gene (MESR3) was overexpressed in C > M, and none in C > B. Overall, the C > B and C > M comparisons flagged fewer GO terms (see discussion).

Regarding the induction of genes associated with the IIS-TOR pathways, insulin-like receptor-like (InR1), Cdk4, and Pdk1 were significantly upregulated in C > P. InR1 and Pdk1 were also upregulated in C > B and C > M albeit not significantly, while Cdk4 was significantly upregulated in C > B but not in C > M.

#### Effects of nutrition in Nosema-infected bees

The GO analysis of expression of the proteinaceous treatments comparatively to carbohydrates revealed larger upregulatory effects of pollen feeding (PN > CN) than Bee-Pro (BN > CN) and MegaBee (MN > CN), thus repeating the observations in healthy animals (Additional file 4). MegaBee-fed bees upheld certain metabolic pathways but inhibited expression (‘chromatin silencing’). Similarly to healthy bees, Bee-Pro effect was minimal, with a single process (‘ion transport’) upregulated in the infected bees. For all proteinaceous diets, many DEGs did not enrich particular bioprocesses (respectively, 28, 10, and 34 genes in PN > CN, BN > CN and MN > CN; DAVID 6.7).

Induction of bioprocesses due to malnutrition in infected bees was more evident comparatively to pollen feeding (CN > PN) than Bee-Pro (CN > BN) and MegaBee (CN > PN), notably stimulating 11 genes associated with response to starvation (Ak6, Mat89Ba, CG8038, l(2)k09022, CG9422, CG12325, CG30349, Atg16, CG14057, Gnat and Dicer-1). More enriched bioprocesses were also obtained in CN > PN (Additional file 5, Fig. 3).

**Fig. 3:**
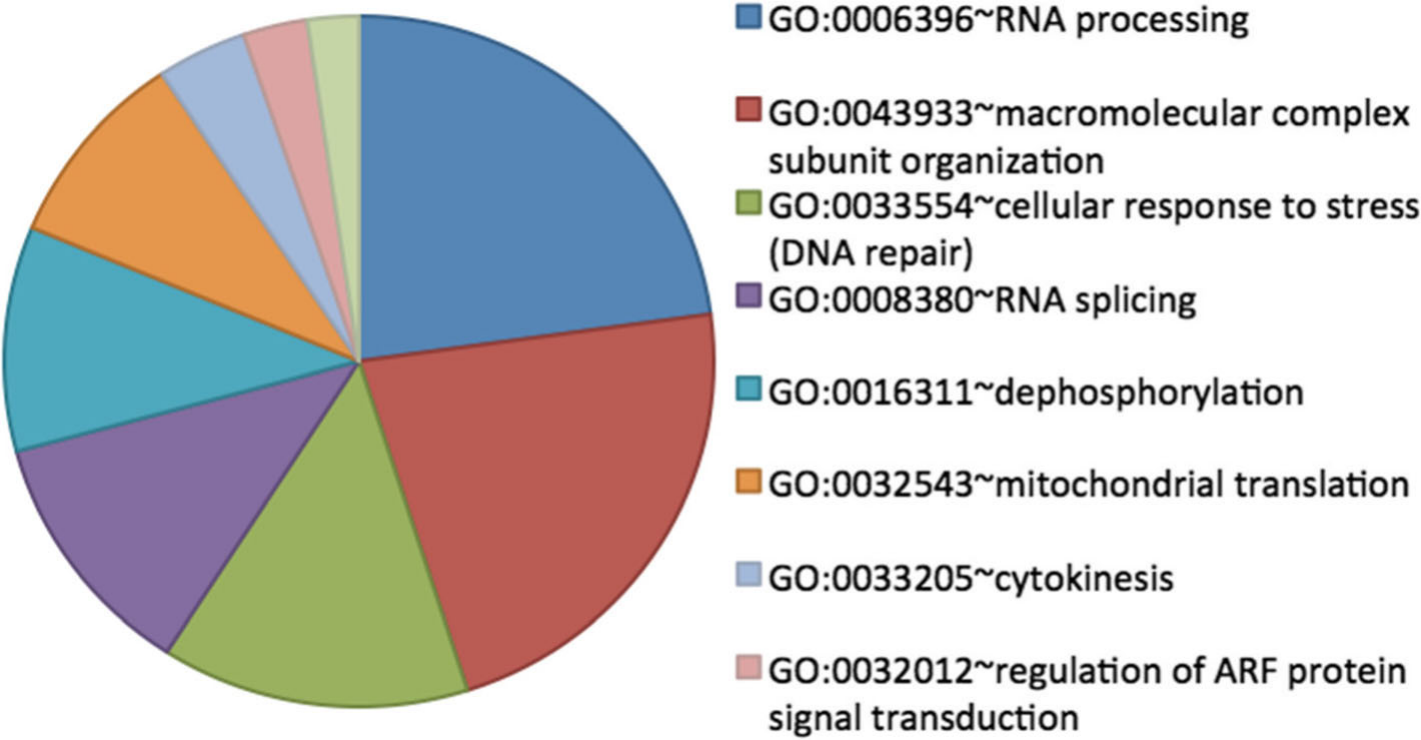
Bioprocesses upregulated under poor diet feeding compared to pollen in presence of Nosema (CN > PN). Pie chart shows the percentage of genes involved in the GO-terms

#### Overlap of nutritional effects

The present work provided several enriched bioprocesses overlapping with a previous transcriptome analysis of different conditions associated with large nutrient stores in honey bee [27]. Key aspects such as type of nutrition, type of enriched bioprocesses and their direction of regulation (Additional files 6 and 7) coincided between both studies.

Within each diet in this work, we identified the DEGs that overlap in the healthy and infected treatments (PN vs. CN/ P vs. C, MN vs. CN/ M vs. C and BN vs. CN/ B vs. C) to detect conserved diet effects in both infection statuses. The overlaps were all significant (Additional file 8), proving they are due to diet effect rather than chance.

**Fig. 4:**
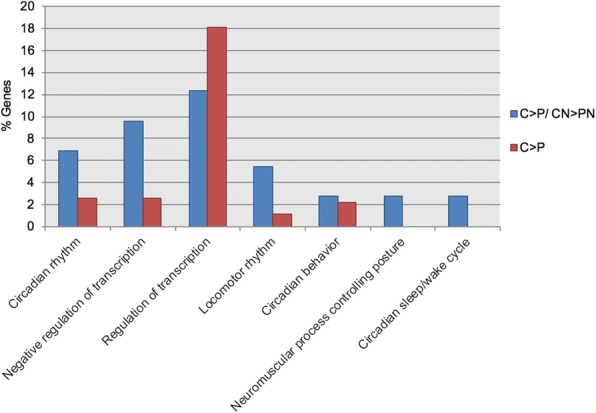
Histogram representing bioprocesses upregulated under poor diet feeding. In blue are GO-terms resulting from the analysis of the overlap between carbohydrates and pollen in absence and presence of *Nosema* (C > P/ CN > PN), and in red are terms upregulated under carbohydrates feeding compared to pollen (C > P). GO-terms with *p* < 0.05 are illustrated

The concordance in the direction of expression was highly significant except for BN vs. CN/ B vs. C (Table 5). Regarding upregulation by rich diet, there was no overlap in BN > CN/ B > C, and few known genes in MN > CN/ M > C and PN > CN/ P > C. The overlapping upregulatory effect by carbohydrates comparatively to Bee-Pro or MegaBee (CN > BN/ C > B and CN > MN/ C > M) was minimal (Table 5). Contrarily, the upregulated overlapping genes by carbohydrates in the pollen comparisons (CN > PN/ C > P) involved a high number of DEGs (Additional file 8) and enriched processes, which almost entirely were also upregulated in C > P (Fig. 4).

**Table 5:** Concordance in direction of regulation of overlapping gene lists

A) Rich diet overlap between presence and absence of *Nosema*	PN vs CN	P vs C				
		Total		Up	Down	Significance
		131	Up	7	0	X-squared = 85.458 *p*-value < 2.2e-16
			Down	2	122	
	BN vs CN	B vs C				
		Total		Up	Down	Significance
		11	Up	0	5	X-squared = 0.090909 p-value =0.763 *
			Down	0	6	
	MN vs CN	M vs C				
		Total		Up	Down	Significance
		21	Up	15	0	X-squared = 8.4077 p-value = 0.003736
			Down	2	4	
B) Overlap between rich diets in healthy bees	P vs C	B vs C				
		Total		Up	Down	Significance
		64	Up	5	0	X-squared = 50.86 p-value = 9.877e-13
			Down	0	59	
	P vs C	M vs C				
		Total		Up	Down	Significance
		56	Up	5	0	X-squared = 44.377 p-value = 2.708e-11
			Down	0	51	
	B vs C	M vs C				
		Total		Up	Down	Significance
		61	Up	22	0	X-squared = 56.74 p-value = 4.974e-14
			Down	0	39	
C) Overlap between rich diets in *Nosema* infected bees	PN vs CN	BN vs CN				
		Total		Up	Down	Significance
		117	Up	4	0	X-squared =88.674 p-value = < 2.2e-16
			Down	0	113	
	PN vs CN	MN vs CN				
		Total		Up	Down	Significance
		90	Up	0	0	X-squared = 67.6 p-value < 2.2e-16
			Down	6	84	
		MN vs CN				
	BN vs CN	Total		Up	Down	Significance
		44	Up	1	0	X-squared =10.494 p-value =0.001197
			Down	0	43	

Overlapping genes are sorted according to direction of regulation. PN vs. CN, BN vs. CN and MN vs. CN are, respectively, pollen, Bee-Pro and MegaBee diet treatments with *Nosema*. P vs. C, B vs. C, and M vs. C are, respectively, the same diet treatments in healthy bees. Chi-square tests with Yates correction were performed to determine if pattern of direction bias is statistically consistent between the considered two conditions. Asterisk (*) indicates lack of pattern consistence

We also examined the significance of overlap between rich diets in healthy bees (P vs. C/ B vs. C, P vs. C/ M vs. C and B vs. C/ M vs. C) (Additional file 8). The direction of expression analysis revealed a complete correspondence for all genes, and a larger number of upregulated transcripts overlapping between Bee-Pro and MegaBee (B > C/ M > C) than with pollen (P > C/ B > C and P > C/ M > C) (Table 5). Regarding the overlapping upregulatory effect by poor diet, C > P/ C > B generated the highest number of DEGs (down-overlap, Table 5), but also enriched bioprocesses (Additional file 9), of which some were also enriched independently in each diet (C > P and C > B).

The numbers of DEGs overlapping between rich diets in *Nosema*-infected treatments (PN vs. CN/ BN vs. CN, PN vs. CN/ MN vs. CN and BN vs. CN/ MN vs. CN) were significant (Additional file 8). The direction of regulation showed perfect conformity in PN vs. CN/ BN vs. CN and BN vs. CN/ MN vs. CN, and was highly concordant for PN vs. CN/ MN vs. CN with only 6 genes showing an opposite direction of regulation (Table 5). The overlaps involved few upregulated genes, with PN > CN/ BN > CN being the only comparison where genes of known function were recorded. Upregulation under carbohydrates feeding in CN > PN/ CN > BN produced the largest number of DEGs (down-overlap, Table 5), and two enriched bioprocesses (Additional file 9).

#### Effects of nutrition on immunity

In the CN > PN comparison, pivotal players of a Toll-mediated antifungal response were stimulated, including sph (SP10/ GB49440, FC = − 2.7), nec (serpine-1/GB46970, FC = − 2.5), TUB (GB51427), cactus (GB53302), Helicase 89B, PGRP-S3, and Apisimin (GB53576). In the CN > BN and CN > MN comparisons, these genes showed the same directional bias, but the changes did not satisfy the *p*-value or FC cutoff criteria.

**Fig. 5:**
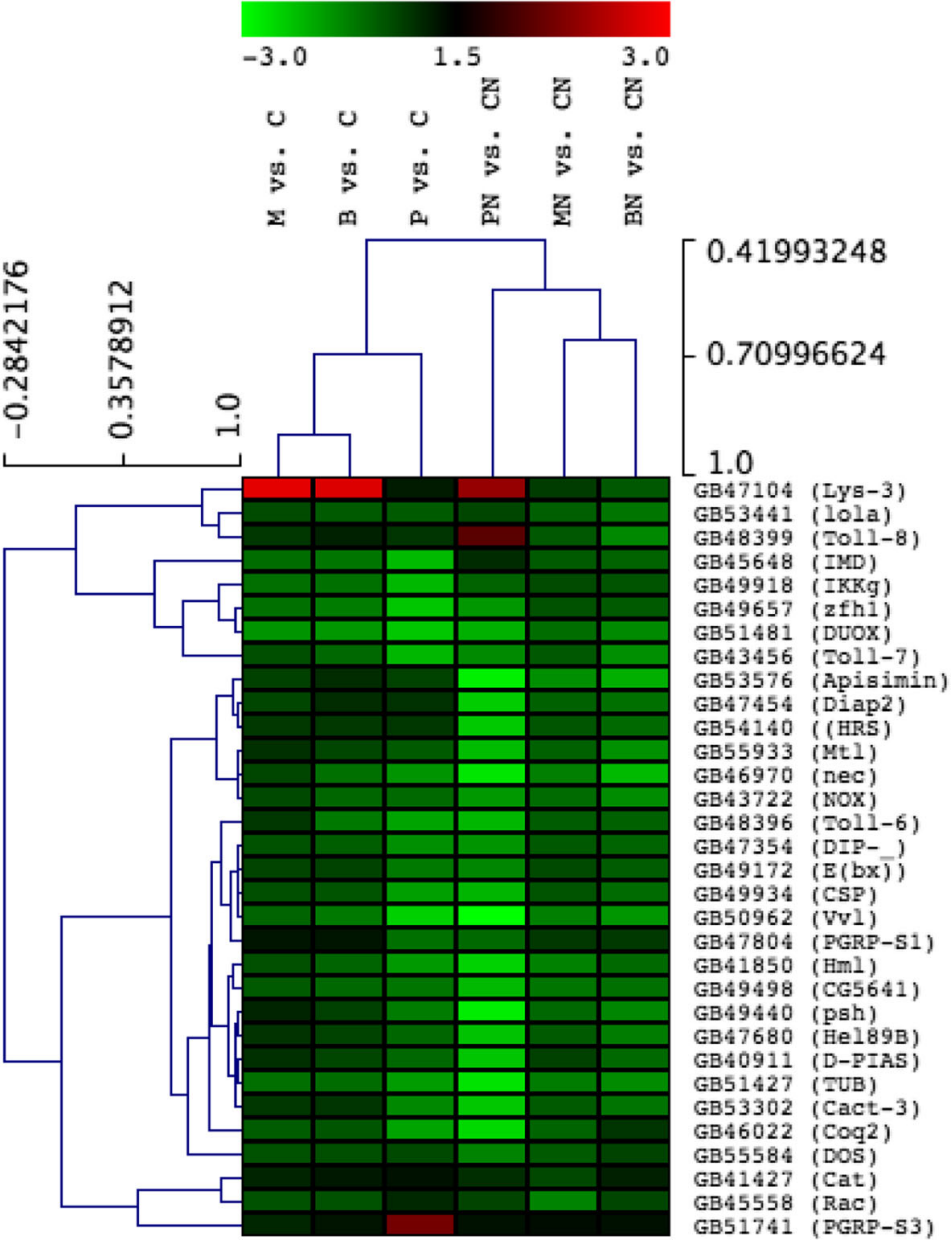
Hierarchical cluster analysis of DEGs involved in immunity and defense in honey bee or Drosophila. Genes significantly influenced at least in one treatment are represented. Considered effects are rich diet feeding, pollen (P vs. C), Beepro (B vs. C) and MegaBee (M vs. C) compared to carbohydrates, and the effects under the same rich diets in presence of Nosema, respectively PN vs. CN, BN vs. CN and MN vs. CN

**Fig. 6:**
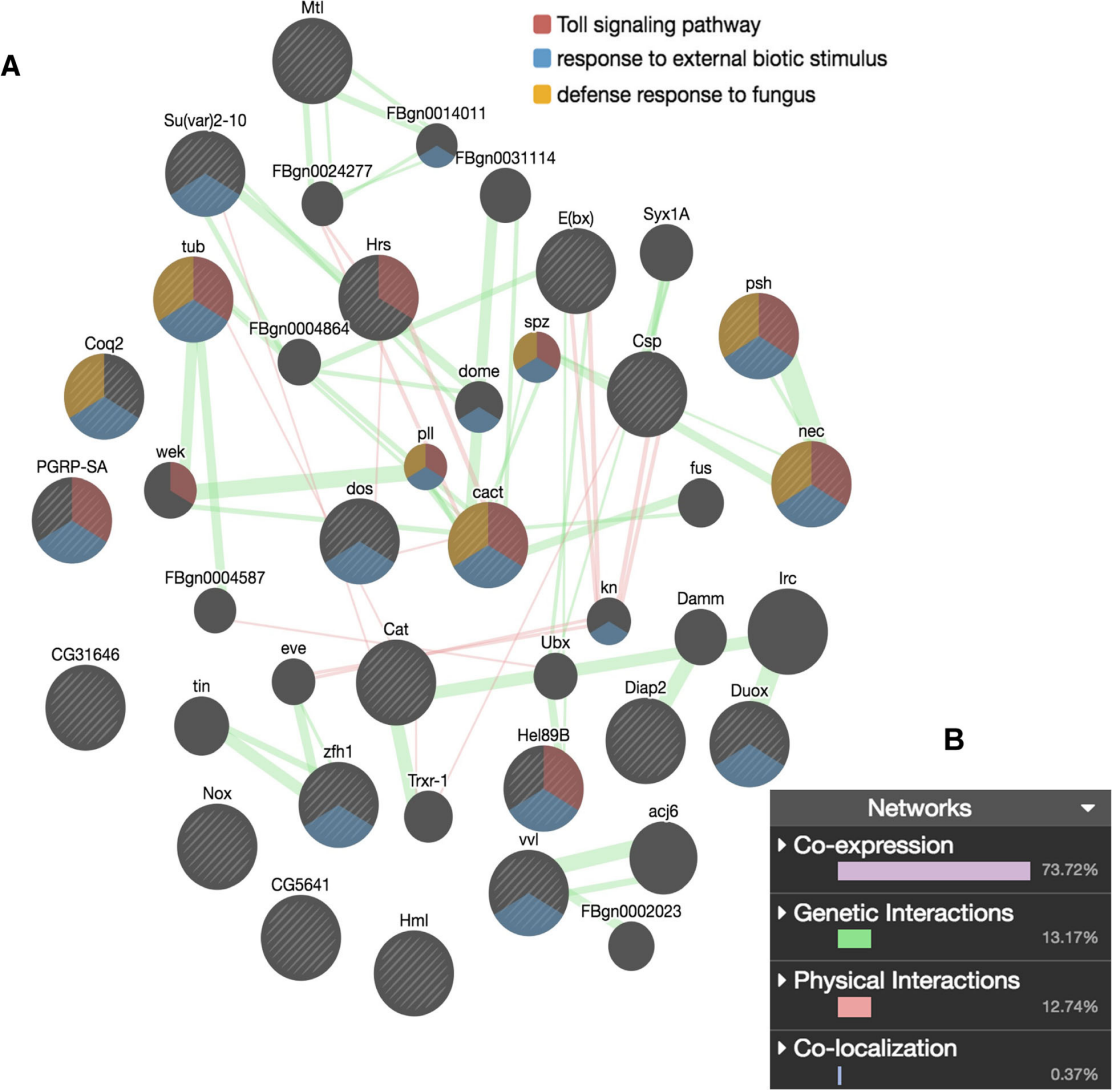
Gene network of immune/defense genes triggered under carbohydtrates feeding in Nosema-infected bees (CN > PN). **a** Network generated using GeneMania; stripped circles are the Drosophila ortologs of DEGs uncovered in the present study; plain black circles are genes uncovered from Genemania database. Physical and genetic interactions between genes are represented by pink and green connections respectively. Some of the significant pathways in relation to Drosophila genome are colored according to above legend. **b** Types of the network gene interactions and their corresponding percent are represented

We uncovered 32 potential immune/defense DEGs (Fig. 5), when screening the diet treatments (P vs. C, B vs. C, M vs. C, PN vs. CN, BN vs. CN and MN vs. CN) for known *A. mellifera* immune/defense genes [73, 74] and *Drosophila* orthologs with immune/defense functions (flybase). The upregulation under rich diets feeding in uninfected bees (P > C, B > C and M > C) and infected bees (PN > CN, BN > CN and MN > CN) was minimal (up to 3 genes). Regarding upregulation under poor diet feeding in healthy bees, the comparisons to Bee-Pro (C > B) and MegaBee feeding (C > M) led to few DEGs, while the comparison to pollen (C > P) uncovered a slightly larger number of DEGs (7 genes). The upregulation under poor diet feeding in infected bees revealed more DEGs (21 genes) compared to pollen (CN > PN) than compared to the substitutes. Because the PN vs. CN comparison yielded the most immune-related DEGs (22 genes), it was further examined using a gene network analysis, with *D. melanogaster* genome as the reference background (Fig. 6). The network analysis uncovered that the 22 potential immune/ defense genes differentially regulated in the abdomen of infected bees fed pollen compared to carbohydrates (PN vs. CN) were interconnected with 74% co-expression (same tissue) and 26% genetic (gene level) or physical interactions (protein level). Of all genes, 68% (15 genes) were interconnected functionally, either genetically or physically, in a single pathway. Notably, in this pathway, all genes were upregulated by poor diet (CN > PN) but the antioxidant catalase that was upregulated by pollen feeding (PN > CN). Importantly, with *Drosophila* genome as the reference, the immune gene list was enriched in bioprocesses that are evidently relevant to fungus infection, especially with an enriched Toll signaling pathway (Table 6).

**Table 6:** Gene network analysis of immune expression

Interaction	%	Bioprocess	FDR	# Genes
Co-expression	73.72	Positive regulation of antimicrobial peptide biosynthesis	7.90e-7	5/ 28
Genetic	13.17	Defense response to fungus	8.15e-6	5/ 52
Physical	12.74	External biotic stimulus	1.23e-11	12/ 288
		Toll signaling pathway	1.30e-9	7/ 47

Immune and defense genes differentially expressed in infected bees fed carbohydrates instead of pollen (PN vs. CN) was analyzed in GeneMania. Types of interactions in the resulting network are described with their percentage. Enriched defense processes were uncovered, of which a select number are described. Gene accounts and FDR were calculated based on the *Drosophila* genome

### RT-qPCR assays

**Fig. 7:**
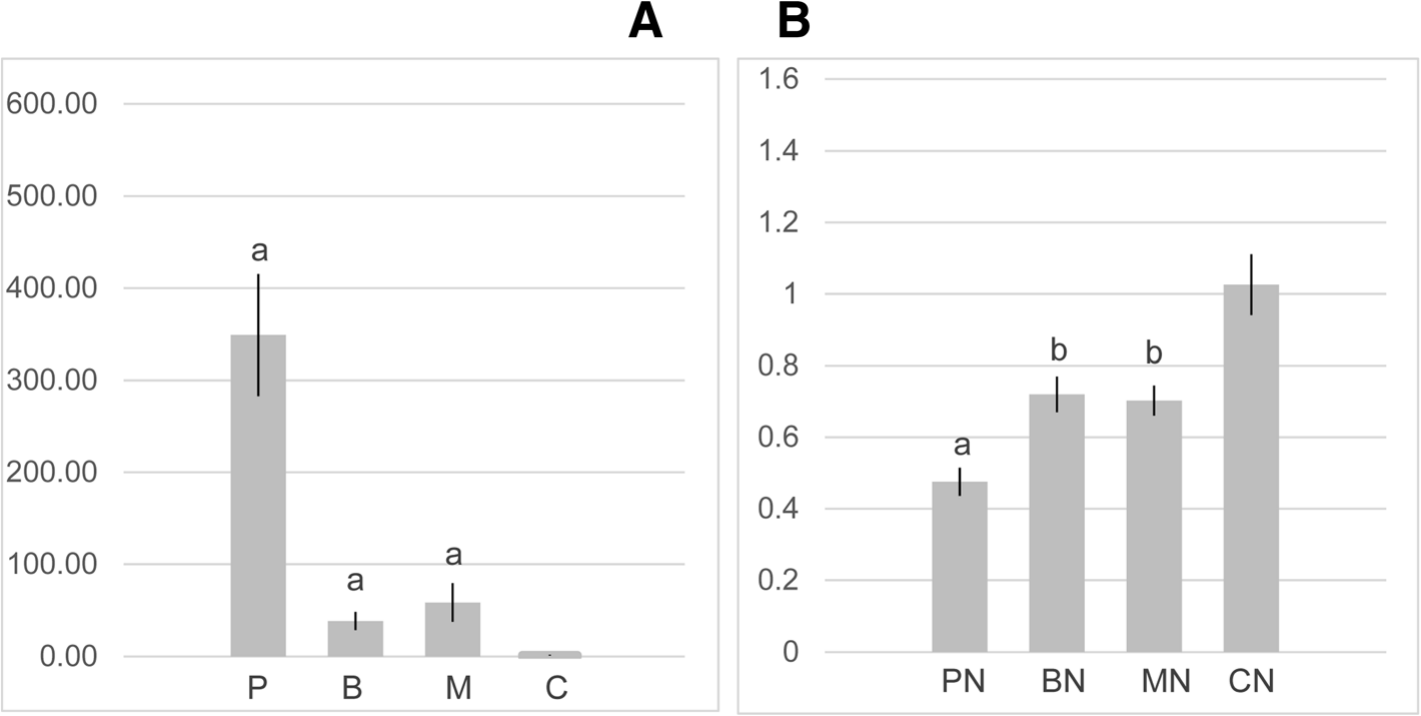
Vitellogenin (Vg) and Duox relative expression measured by qPCR. **a** Healthy bees were fed pollen (P), Bee-Pro (B), MegaBee (M) or carbohydrates (C; [control]); regulation of Vg in the proteinaceous treatments was determined compared to C; significance levels are indicated with letters b (*p* < 0.01) and a (*p* < 0.001); error bars represent the standard error. **b** *Nosema-*infected bees were fed pollen (PN), Bee-Pro (BN), MegaBee (MN) or carbohydrates (CN); Duox regulation by the rich diets was determined compared to CN; significance levels are indicated with letters b (p < 0.01) and a (p < 0.001); Wilcoxon’s test performed when p < 0.001; error bars represent the standard error

For both genes, Vg and Duox, and in all treatment comparisons, qPCR results agreed with RNA-seq in terms of direction of regulation and diet classification. The RNA-seq strategy had revealed Vg was highly expressed, but with insignificant *p*-value, in healthy bees fed pollen (P > C: FC = 27.76, *p* = 0.17), followed by MegaBee (M > C: FC = 11.74, *p* = 0.30) and Bee-Pro (B > C: FC = 6.94, *p* = 0.11). Results of the qPCR analysis confirmed Vg overexpression under all rich diets and in the same order (Fig. 7a), and with significant *p*-values (P > C: *p* = 4.114e-05; M > C: p = 4.114e-05; B > C: *p* = 8.227e-05). Similarly, the results of Duox expression analysis via qPCR and RNA-seq were in agreement, showing upregulation under poor diet feeding was more evident in CN > PN comparison than CN > BN and CN > MN (Fig. 7b). However, by contrast to RNA-seq, p-values attributed to the latter two comparisons were significant in the qPCR approach.

## Discussion

### Protein content

From the protein analysis results, it appears that in terms of quality, pollen has greater amounts of protein that is more readily digested by bees than either pollen substitute we tested. In fact, although diet quality is commonly associated to protein content, the nutritional value to an organism depends on more than this single parameter. For example, the nutritional value of pollen to bees is primarily defined by its absolute and relative content of essential amino acids [75]. Similarly, the protein titer of the hemolymph is a good indicator of a diet’s value to bees [76]; nevertheless, it is only one of many such indicators, making conduction of several bioassays measuring different parameters necessary to determine the value of a given diet to honey bees [77]. Some examples include hypopharyngeal glands development [24, 62, 78] and protein content [79], ovarian development [24, 80], longevity [81, 82], and fat body weight [83].

### Genome-wide regulation

The honey bee tendency to genome-wide upregulation under conditions of stress observed herein was also reported in *Escherichia coli* cells, which systematically increased the number of expressed genes as substrate quality declined [84]. This upregulation was seemingly contradictory to the expected slow growth in limiting conditions. However, rRNA synthesis that correlates to growth rate [85] decreased proportionately with reduced growth. These results implied that in response to the nutrient cues, a bias toward upregulation was possibly triggered via global patterns of RNA polymerase (RNAP) distribution. In fact, in *E. coli*, under optimal growth conditions, only few RNAP molecules are dedicated to transcribing 99% of the genes, while the majority of RNAP molecules transcribe the remaining 1% which encode rRNA and tRNA [86]. Under suboptimal conditions, few RNAP molecules transcribe rRNA and tRNA genes, thus inciting a reprograming of the transcription machinery in such a way that these genes are inhibited and others are activated.

### Differential regulation of gene expression

#### Upregulatory effects of rich diets in healthy bees

The pollen diet incited more rich nutrition-related bioprocesses than both pollen substitutes. Stimulation of ‘proteolysis’ in pollen-fed bees corroborates the above discussed protein analysis results, and may reflect digestibility and degradation within the cell to procure amino acids for synthesis of new peptides. Enrichment of purine ribonucleotides biosynthesis mirrors an expected effect on anabolism, since these compounds have a wide variety of cell functions. This enrichment also might be due to a specific but unknown growth demand similarly to well-fed *Drosophila* females, which upregulated ribonucleotides synthesis probably in relation to increased egg production [87]. On the other hand, the upregulation of acetyl-CoA metabolism in P > C did not concord with the downregulation of energy pathways in bees fed rich vs. poor diet that mirrors the metabolic profile of nurses vs. foragers [27]. One tangible explanation is a possible increase of acetyl-CoA metabolism due to excess of amino acids in pollen. Indeed, in animals such as mice [88] and rat [89] fed proteinaceous diets, the surplus of amino acids not used in biosynthesis cannot be stored, and is instead converted into acetyl-CoA, a major metabolic intermediate of the tricarboxylic acid cycle. Therefore, it might be that in bees under nutritional excess, there is increase of acetyl-CoA metabolism as well as vitellogenin storage (Fig. 7a) to utilize the surplus of macronutrients.

Lipid metabolism is indicative of response to rich nutrition [27, 28], and was the only significant general aspect of metabolism affected by all three proteinaceous diets tested in this study. In bees fed MegaBee, other upregulatory aspects of rich nutrition resided in the enrichment of cell cycle bioprocesses (e.g. ‘cytoskeletal organization and biogenesis’ and ‘mitotic spindle organization’) suggesting guts tissue homeostasis, and ‘cellular amino acid biosynthesis’ (*p* = 0.051; the latter bioprocess being the child term of ‘carboxylic acid biosynthesis’) that may reflect peptide biosynthesis.

#### Upregulatory effects of poor diet in healthy bees

Bees fed carbohydrates compared to pollen (C > P) showed increased expression of bioprocesses associated with suboptimal nutrition. Downregulatory mechanisms of transcription regulation, macromolecule biosynthesis, and translation were all enriched denoting an overall repression of the main drivers of growth. Consistent with this result, C > P stimulated hippo signaling, a conserved pathway that regulates growth principally by restraining cell proliferation and promoting apoptosis. Knowing that upregulatory mutations of hippo signaling cause dramatic changes in organ size, mostly the liver [90], our result implies a possible role in curbing growth in the honey bee abdomen. Developmental processes were widely elicited, suggesting reuse of such functions is linked to foraging transition. In fact, commonalities between development and phenotypic plasticity were signaled in foragers versus nurses [91], and poor diet versus pollen [27]. Moreover, ‘behavior’ and ‘adult behavior’ bioprocesses were upregulated suggestive of behavioral maturation and reinforcing the assumption of a possible transition to foraging in bees fed deficient nutrition.

Another important aspect of poor nutrition, evident in C > P, is the modulation of the ‘circadian rhythm’ bioprocess. This infers the abdominal peripheral clocks may be altered to adapt the metabolic demands to environmental stress. Although food was provided ad libitum, ‘feeding behavior’ was also altered, possibly due to changes of feeding activity imposed by poor nutrition. These results are in accord with a meta-analysis study (19 tissue types; most frequently liver tissues), which compared caloric restriction to ad libitum feeding in mammals [92]. In the study, ‘rhythmic process’ and ‘circadian rhythm’ were among the most upregulated bioprocesses, and two of the top 10 upregulated markers were circadian clock genes. The emergence of peripheral clocks as players in the adjustment to poor nutrition is not surprising since these clocks are dependent on the feeding cycle [93, 94].

Also, bees fed poor diet compared to pollen (C > P), in agreement with previous reports [27], showed amplified cell communication (e.g. intracellular and cell-cell signaling). The ability of cells to communicate environmental cues, including nutrients availability, is the basis for adapting and maintaining vital functions. Thus, cell signaling, especially since it comprises the response to starvation gene Ac76E, might be due to the imposed suboptimal conditions.

Regarding the comparisons of carbohydrates feeding to the substitutes, C > B showed enriched cell communication (‘signal transduction’; fewer terms than C > P), regulation of transcription (5 out of 8 genes overlapping with C > P), and development (‘multicellular organism development’). These results denote the richness of Bee-Pro compared to carbohydrates only diet, however, the fact that these bioprocesses were more clearly enriched in C > P, and the higher number of altered bioprocesses in C > P reflects the superior nutritional value of natural versus artificial diet. In the case of the comparison to MegaBee, the paucity of enriched bioprocesses in C > M denotes that MegaBee is similar in many respects to the carbohydrate diet and implies that on certain aspects, this diet has lesser nutritional value than pollen and Bee-Pro.

A final interesting aspect of enriched bioprocesses in response to poor nutrition resides in the alteration of maturation cues (development, behavior and adult behavior) apparent only in comparison to pollen. This result indicates that the disparity between poor and rich diet causing these effects depends on rich nutrition composition and not on rich diet as a whole. This outcome also suggests pollen as better for bee nutrition.

With regard to the influence of nutrition on the IIS-TOR pathways in bees, a previous report indicated a nurse-like downregulatory pattern when bees were fed rich diet, and a forager-like upregulatory pattern when fed poor diet [27]. For example an upregulation of the insulin-like receptor (InR1) was reported in bee nurses aged 4–6 days fed sugar instead of pollen, in both brain and abdomen [71]. The 14-days old bees fed poor diet instead of pollen in this study showed a significant upregulation of INR1 confirming the expected poor nutrition response to carbohydrates compared to pollen. The same upregulatory effect was observed when carbohydrates-fed bees were compared to the substitutes, albeit without statistical significance. It is noteworthy that under poor diet feeding (C > P), although IIS-TOR pathways genes were upregulated in the abdomen, translation as whole was repressed as seen above.

#### Upregulatory effects of rich diet in Nosema-infected bees

Despite *Nosema* infection, animals fed pollen (PN > CN) maintained the upregulatory effects and enrichment of certain metabolic functions observed in the healthy treatment, while infected bees fed Bee-Pro (BN > CN) or MegaBee (MN > CN) showed less favorable effects. Pollen upheld the metabolism of amino acids (histidine, tryptophan), and also that of lipids (6 DEGs; ‘oxidation-reduction’ bioprocess). The activation of catalase (‘oxidation-reduction’ bioprocess), a key component of response to oxidative stress, shows that pollen-fed infected bees not only upregulated some aspects of rich nutritional status, they also combated disease stress. The above considerations imply pollen upregulatory effects under *Nosema* parasitism enabled bees to maintain function while reducing stress.

#### Upregulatory effects of poor nutrition in Nosema-infected bees

Consistent with poor nutritional status, malnourished-infected bees compared to infected bees fed pollen (CN > PN) overexpressed several stress-related bioprocesses.

The broad ‘cellular response to stress’ bioprocess was triggered, and included ‘DNA damage stimulus’ and ‘DNA repair’, thus reflecting the induction of genetic repair mechanisms. Since these bioprocesses were not enriched in C > P, it is conceivable that a combination of disease and nutritional stress leads to elevated DNA damage. Amino acid starvation possibly increased parasite sensitivity, while the main driver of DNA damage might have been *Nosema*, since microsporidia cause DNA damage to host cells [95], notably to gastrointestinal cells (increasing mutations rate) [96].

The overexpression of the ‘RNA processing’ bioprocess in CN > PN conforms to previous reports in similar nutritional conditions [27]. This outcome is not surprising since modulation of RNA processing, including splicing and alternative splicing (AS), is involved in stress tolerance [97–100].

Inadequate nutrition might have been a regulator per se as ‘RNA processing’ included response to starvation genes (l(2)k09022, CG14057 and CG8038). This echoes mammalian cell cultures, which when lacking amino acids, activated pathways controlling transcription and RNA processing from chromatin structure to translation initiation [101]. In animals, AS plasticity is crucial to stress tolerance, since it produces more efficient stress response isoforms [102], including heat shock [103, 104] and genotoxicity [105, 106]. In this work, RNA processing and spliceosome machinery upregulation indirectly suggests increased AS. The AS regulatory proteins (control of gene-specific splicing [107]), which enrich ‘RNA processing’, also support such an assertion.

Nuclear transport, especially nuclear import, is downregulated by cellular stress [108, 109], including starvation [110]. In fact, cytoplasm-nucleus trafficking is a highly regulated pathway and would be affected if cellular homeostasis were compromised. The overexpression of the bioprocess ‘negative regulation of protein import’ in CN > PN implies suboptimal nutrition aggravated by disease adversely impacts the effectiveness of molecular trafficking. In addition, there might have been active interference by *Nosema* since microsporidia subvert normal host cell processes [111].

In CN > PN, the upregulation of the bioprocess ‘macromolecular complex subunit organization’ mirrors a similar result under carbohydrate diet and vitellogenin knockdown [27]. In the present study, this bioprocess involves genes, which in *Drosophila* or mammals, are associated with immunity, pathogen invasion, disease, apoptosis, stress response, and cell senescence (Additional file 10). Interestingly, some genes (Taf4, Trf, Taf5, Nlp and Tfb4) enriching this bioprocess are associated with human HIV infection and life cycle. ‘RNA biosynthetic process’, which was also enriched, comprised genes functioning in defense response to fungus, DNA repair, immunity, and HIV or influenza life cycles (Additional file 10). The activation, in the *Nosema*-infected bees, of genes that were identified in human infectious diseases suggests a possible role in *Nosema*-honeybee interactions. In addition, the overexpression of immune and stress response genes in CN > PN denotes a pronounced disease in the context of malnutrition. Indeed, knowing the immune response is deployed minutes after infection, at day-7 post-infection, pollen-fed bees possibly tolerated the infection while poorly fed bees did not.

‘Mitochondrial translation’ was stimulated in CN > PN, but not under malnutrition alone (C > P) since none of the 17 mitochondrial ribosomal proteins were differentially expressed in the latter comparison. This conforms to the mingled effects of poor nutrition and parasitism. For example, amino acids-deprived human cell cultures, which energy needs were satisfied, still geared mitochondrial metabolism towards amino-acid consumption instead of preservation [112]. This seemingly inefficient response, in the latter report and in our study, might be a way to hinder cytosolic translation that drives growth. Regarding microsporidia-related stress, these amitochondriate parasites might have exploited the host cell oxidative metabolism to support their own needs as previously observed [113–115].

(CN > MN) overexpressed ‘translation’ bioprocess. Although ‘mitochondrial translation’ was not upregulated per se, half of the ‘translation’ genes were mitochondrial ribosomal proteins. Interestingly, other genes include Tpc1 involved in mitochondrial transport, and RpS16 associated in humans with viral mRNA translation, and the influenza infection/life cycle. Thus, seemingly poor nutritional status enables *Nosema* to exploit the translational mechanisms in the host cell, especially that of mitochondria. However, although it is apparent against MegaBee, it is compared to pollen that this aspect is most evident, as shown by the more marked activity of the mitochondrial translation apparatus.

Infected animals fed carbohydrates compared to Bee-Pro (CN > BN) upregulated ‘chitin-based embryonic cuticle biosynthesis’, enriched with chitin or chitin-based cuticle biosynthesis genes, including dib, which is also involved in the midgut development [116]. The stimulation of chitin biosynthesis in the honey bee abdomen in stress conditions is not unexpected. In insects, the chitinous matrix lining the gut mediates immunity by acting as a barrier that prevents pathogens from direct contact with the epithelium [117–119].

In CN > BN, ‘oxidation-reduction’ was also enriched, and involved genes consistent with a metabolism countering *Nosema*-caused cellular stress in the gut. Such genes are Henna (phagocytosis), NADPH oxidase (Nox; gut antimicrobial activity through production of reactive oxygen species [ROS] [120]), Alr (tissue regeneration) and CG14221 (cell redox homeostasis). Upregulation of oxidation-reduction was also seen in bee infected with *N. ceranea*, possibly due to an enhanced generation of ROS in response to the infection [121].

The components of the gut defense response uncovered in this study, namely chitin biosynthesis, ROS production, redox homeostasis, and tissue renewal are overall supported in *Drosophila*. In this insect, the response is comprised of four steps: 1) physical barriers (i.e. peritrophic matrix); 2) production of ROS; 3) secretion of antimicrobial peptides (AMPs) into the hemolymph; and 4) epithelium renewal in response to gut damage [122].

### Overlap of nutritional regulatory effects

#### Within-diet overlap in presence and absence of Nosema

The overlapping-upregulated DEGs in the MegaBee feeding treatments (MN > CN/ M > C) comprised only few genes of known function, which were mostly related to mitotic and meiotic processes (Klp3A, Ack, CG2852 and Cep135), alluding to probable cell proliferation with MegaBee feeding regardless of infection status. The known overlapping-upregulated DEGs in the pollen feeding treatments (PN > CN/ P > C) involved genes functioning in proteolysis (SP22 and SP36), lipid transport and metabolism (Rfabg, CG6300, pudgy and Aldh) consistent with a response to rich nutrition that is maintained in the presence of *Nosema*.

The upregulatory effects of the carbohydrate feeding treatments when compared to the pollen feeding treatments (CN > PN/ C > P) comprised a large number of DEGs, and enriched bioprocesses associated with repressed transcription, adaptative circadian rhythm, altered behavior, and reused developmental mechanisms. These bioprocesses, which were also observed in C > P, show that nutritional stress response involved similar mechanisms in both infection statuses, but possibly more moderately under infection (GO-terms not enriched in CN > PN), perhaps to harness resources for response to the *Nosema* infection (e.g. DNA repair). The overlap also overexpressed ‘response to oxidative stress’ (*p* = 0.0822), probably due to *N. apis* as previously observed with *N. ceranea* infection of bee gut [121]. Also, this bioprocess included the gene Oamb which is responsive to starvation [123], suggesting that aside from microsporidia, malnutrition incites oxidative stress. Inadequate nutrition stimulated adenylate kinase 6 (Ak6), which is involved in the stress-induced pathways, NF-κB (cell survival control), and p53 (genotoxic/non-genotoxic stress, starvation response [124–126]. Similarly to our study, Ak6 was previously implicated in the response to starvation [100].

#### Between-diets overlap in healthy bees

The complete correspondence in direction of regulation of the DEGs overlapping between the rich diets (P vs. C/ B vs. C, P vs. C/ M vs. C and B vs. C/ M vs. C) indicates a clear-cut common response to proteinaceous nutrition, setting the proteinaceous diets apart from the carbohydrates-only diet. Moreover, in all comparisons, numerous genes (about half) have an unknown function, implying that many aspects of the molecular nutritional response are still unknown. The larger number of common DEGs in bees fed the two substitutes than in pollen highlights the major differences separating the natural diet from the artificial substitutes. Finally, in response to malnutrition, ‘regulation of transcription’ was clearly overexpressed in the triple comparison to rich diets (C > P/ C > B/ C > M). Additionally, ‘multicellular organism development’ overexpression in C > P/ C > B, reflected the outcomes of the single diets comparisons (C > P and C > B). These results further support the concept that regulation of gene expression and repurposing of developmental genes are key processes of response to amino acid starvation in adult honey bee.

#### Between-diets overlap in infected bees

The overlapping-overexpressed DEGs between pollen and Bee-Pro treatments in infected bees (PN > CN/ BN > CN) included melittin (active antimicrobial compound of bee venom), and genes involved in immunity or host-pathogen interactions; these are the transmembrane transport protein CG11739 that in humans functions in HIV interactions, Rfabg associated with lipid transport and scavenging by class B receptors, and PGRP-SA that regulates Toll signaling. These results suggest common grounds of defense mechanisms in bees fed pollen and Bee-Pro, but not MegaBee.

The overrepresentation of ‘oxidation-reduction’ bioprocess in CN > PN/ CN > BN overlap supports the idea that under nutritional stress, defense against *Nosema* includes the steps: 1) countering microbes through superoxide release (Nox); 2) combating toxicity by breakdown of the excess superoxide (CG31028); 3) ensuring redox homeostasis at the cell level (CG14221); 4) promoting midgut development (dib). The lack of these mechanisms in overlaps involving CN > BN implies similarities of MegaBee to the carbohydrate diet.

### Nutritional regulation of immunity

A high expression of immunity was expected in a rich nutritional status (especially with *Nosema* infection), contrarily to an inhibited expression in a poor nutritional status. This is because rich nutrition enhances immune functions [127–129], while nutrient deficiencies cause immune dysfunction [130]. However, herein, rich and poor diets had minimal upregulatory effects on immunity expression including when bees were infected. These results are similar to a reported minimal upregulation under pollen feeding and a moderate increase under carbohydrates feeding, even in presence of varroa infection [131]. Other similar reports include a lack of PO (phenoloxidase) response to diet quality in caterpillars [128], and of PO and GST (glutathione-S-transferase) to ameliorated pollen quality or *Nosema* infection in honey bee [22]. The lack of immune overexpression in *Nosema*-infected bees seems to be a general trend observed with *N. apis*, *N apis* and *N. ceranea* co-infection [132], and *N. ceranea* [121]. In the current study, basal constitutive expression of immune genes might be sufficient to mount the initial defense response in case of a pathogen attack, hence the lack of upregulation under healthy and rich nutritional status. In presence of pathogens, the honey bee might rely on different mechanisms to counter the attacks. In infected bees fed pollen (PN > CN) such mechanisms might be reflected by the upregulation of vitamin C (‘ascorbate and aldarate metabolism pathway’), which in humans protects against oxidative stress and has a role in immunity [51, 133, 134] as well as catalase, which has a pivotal role in protection against ROS. In fact, residual ROS has inflammatory effects, and a balance between synthesis and elimination of ROS via antioxidants is necessary to protect the gut, as seen against *N. ceranea* [121]. Furthermore, PN > CN upregulated transcripts involved in gut morphogenesis and development genes (dpp, Zipper and garz), which infers that gut host defense may encompass epithelial renewal [121, 135]. This result agrees with previous observations where *N. apis* disrupted midgut development [132], and *N. ceranea* inhibited tissue renewal [121].

The slightly increased expression of immune/defense genes in CN > PN might reflect an escalated defense response in bees fed carbohydrates due to difficulty overcoming *Nosema*. In fact, defense activation was persistent even during late stage of infection (7 days post infection) relying on antimicrobial peptide biosynthesis (vvl, Diap2, Hel89B) and defense response to fungus (coq2 [136], cact, tub [137], and psh [138]). Moreover, the overexpression of Mtl associated with response to DNA damage [139], and Duox that produces hydrogen peroxide are cues of a mounted defense against a DNA-damaging microsporidium-like pathogen. The induction of Duox hints at its pivotal role in bee gut defense response, mirroring that of *Drosophila* in which this enzyme is a key effector against ingested microbes [120, 135, 140, 141]. In the CN > PN comparison,

the defense response (defense peptides, Toll pathway genes, and Duox) was in concert with the upregulation of cellular stress response, especially DNA repair. The defense response in the context of nutritional stress is even more clear in the overlap CN > PN/ CN > BN as discussed previously.

The significant upregulation of the defense response genes (including Toll pathway genes) in CN > PN but not in CN > BN and CN > MN suggests that, in bees fed the substitutes, the expression of these genes was sufficiently high to not create significant differences with carbohydrates feeding. However, because most of these genes exhibited the same direction of regulation in animals fed the different proteinaceous diets, there may be a comparable yet nuanced immune response in bees fed pollen, Bee-Pro and MegaBee.

### RT-qPCR analysis of vitellogenin modulation

In the honey bee, the yolk lipoprotein vitellogenin, synthetized in the fat body, is a storage protein with multiple functions, including utilization in jelly production [142], promotion of longevity [26, 143] and immunity [144]. Vitellogenin levels are nutritionally modulated; specifically, lack of proteins intake drastically reduces vitellogenin expression [22, 27, 76, 131, 145].

In the present study, as expected, vitellogenin was considerably upregulated in bees fed protein-based diets compared to carbohydrates only. However, Vg overexpression under pollen feeding was considerably higher than bees fed MegaBee or Bee-Pro (more than 290 times greater), possibly reflecting the difference in protein quantity and quality of pollen relative to the commercial diets. In a recent study, varying quality of pollen-based diets was also shown to play a role in vitellogenin expression, since poor-quality seasonal pollen (maize) induced poor nursing physiology notably vitellogenin expression [146].

## Conclusions

Deep insights have been gained into the differences of honey bee genomic response to pollen feeding versus Bee-Pro and MegaBee. The study also provided insight into the nuanced defense response of honey bee to *Nosema* infection when fed pollen instead of pollen substitutes. The most salient conclusion is the advantage pollen diet provides over Bee-Pro and MegaBee in conferring a richer nutritional status to bees, including in presence of a fungal pathogen. Clearly, the superiority of pollen to artificial substitutes cannot be generalized since such claims should be substantiated by further studies involving larger selection of pollen mixtures and substitutes. Also, the data obtained through gene expression should be analyzed further by protein assays to confirm the modulation of the uncovered pathways. Nonetheless, the analysis presented herein supports the hypothesis that a balanced, natural diet allows bees to maintain a healthy metabolism and, in case of disease, provides individuals with a better fitness to mitigate the pathologic stress. Thus, a balanced, natural diet is essential to individuals and, by extension to overall bee colony health.

## Additional files

Additional file 1: **Table S1.** Subset of genes responsive to nutrition quality with or without *Nosema*. List of a select number of genes differentially regulated in at least 2 diet treatments in infected or healthy bees. Genes were chosen based on known function or *Drosophila* ortholog. (XLSX 48 kb)

Additional file 2: **Table S2.** Bioprocesses and pathways upregulated in healthy bees under rich diet. Upregulated bioprocesses (GO-terms) and Kegg pathways in healthy bees under pollen (P > C), Bee-Pro (B > C) and MegaBee (M > C) feeding compared to carbohydrates-only diet. (XLSX 46 kb)

Additional file 3: **Table S3.** Bioprocesses and pathways upregulated in healthy bees under poor diet. Upregulated bioprocesses (GO-terms) in healthy bees under poor diet feeding (carbohydrates-only) compared to pollen (C > P) and Bee-Pro (C > B). (XLSX 48 kb)

Additional file 4: **Table S4.** Bioprocesses and pathways upregulated in *Nosema*-infected bees under rich diet. Upregulated bioprocesses (GO-terms) and Kegg pathways in *Nosema*-infected bees under pollen (PN > CN), Bee-Pro (BN > CN) and MegaBee (MN > CN) feeding compared to carbohydrates-only diet. (XLSX 36 kb)

Additional file 5: **Table S5.** Bioprocesses and pathways upregulated in *Nosema*-infected bees under poor diet. Upregulated bioprocesses (GO-terms) and Kegg pathways in *Nosema*-infected bees under poor diet feeding (carbohydrates-only) compared to pollen (C > P), Bee-Pro (C > B) and MegaBee (CN > MN). (XLSX 92 kb)

Additional file 6: **Table S6.** Bioprocesses upregulated under rich diet overlapping with the study by Ament et al. (2011). In the present study, upregulation in healthy bees under Bee-Pro (B > C), MegaBee (M > C) and pollen (P > C) feeding is represented compared to carbohydrates-only diet; respectively, BN > CN, MN > CN and PN > CN represent the same diets comparisons in *Nosema*-infected bees. Overlapping bioprocesses with the study by Ament et al. (2011) are listed; upregulated treatments in the latter study are nurse vs. forager (N > F), rich diet vs. poor (R > Pr), vitellogenin wild type vs. vitellogenin RNAi (Vg > Vg(−)), and queen mandibular pheromone treatment (QMP) vs. control (Q > Ct). All listed bioprocesses are upregulated in this study (+); bioprocesses that are expressed in the opposite direction in Ament et al. are indicated with the sign minus (−). (XLSX 10 kb)

Additional file 7: **Table S7.** Bioprocesses upregulated under poor diet, overlapping with the study by Ament et al. (2011). Upregulation in healthy bees (no-*nosema*) under carbohydrates-only feeding is represented compared to pollen (C > P), and in presence of *Nosema* compared to MegaBee (MN > CN) and pollen (PN > CN). Overlapping bioprocesses with the study by Ament et al. (2011) are listed; upregulated treatments in the latter study are forager vs. nurse vs. (F > N), poor diet vs. rich (Pr > R), vitellogenin RNAi vs. vitellogenin wild type (Vg(−) > Vg). All listed bioprocesses are upregulated in this study (+); the non-concordant bioprocesses in direction of regulation in Ament et al. are indicated by the sign minus (−). (XLSX 11 kb)

Additional file 8: **Table S8.** a) Gene overlap of pollen and Bee-Pro feeding (P vs. C/ B vs. C), pollen and MegaBee feeding (P vs. C/ M vs. C) and Bee-Pro and MegaBee feeding (B vs. C/ M vs. C) in healthy bees. b) Respectively, P vs. C, B vs. C and M vs. C are healthy bees fed pollen (P), Bee-Pro (B) and MegaBee (B) compared to carbohydrates only (C); respectively, PN vs. CN, BN vs. CN and MN vs. CN are the same diet treatments in *Nosema*-infected. c) Gene overlaps of PN vs. CN/ BN vs. CN, PN vs. CN/ MN vs. CN and BN vs. CN/ MN vs. CN are, respectively, the same diet comparisons in presence of *Nosema*. RF is the representation factor indicating fold enrichment for the overlap, and *p*-value is the overlap statistical likelihood based on a hypergeometric distribution. (XLSX 10 kb)

Additional file 9: **Table S9.** Upregulated bioprocesses resulting from different overlaps of poor diet comparisons. Upregulated bioprocesses, in healthy bees, generated by carbohydrates-only feeding in the overlaps of pollen and Bee-Pro (C > P/ C > B), Bee-Pro and MegaBee (C > B/ C > M), and the triple overlap of pollen, Bee-Pro and MegaBee (C > P/ C > B/ C > M) are listed; also listed are the same comparisons in presence of *Nosema*, respectively, CN > PN/ CN > BN, CN > BN/ CN > MN and CN > PN/ CN > BN/ CN > MN; the comparison CN > PN/ CN > MN indicates overlap of upregulation by poor diet when compared to pollen and MegaBee in infected bees. The upregulated bioprocesses generated by carbohydrates-only when compared to pollen in healthy and *Nosema*-infected bees (C > P/ CN > PN) are also indicated. (XLSX 12 kb)

Additional file 10: **Table S10.** Potential immune/defense genes enriching GO:0043933~macromolecular complex subunit organization. (XLSX 11 kb)

## Abbreviations

AS: Alternative splicing
DEGs: Differentially expressed genes
FDR: False discovery rate
FPKM: Fragments Per Kilobase of transcript per Million fragments mapped
GO: Gene Ontology
HIV: Human immunodeficiency virus
KEGG: Kyoto Encyclopedia of Genes and Genomes
REVIGO: Educe and visualize gene ontology
RNAP: RNA polymerase
RNA-Seq: RNA sequencing
RT-qPCR: Reverse transcription-quantitative polymerase chain reaction
